# Black Phosphorous-Based Surface Plasmon Resonance Biosensor for Malaria Diagnosis

**DOI:** 10.3390/s25072068

**Published:** 2025-03-26

**Authors:** Talia Tene, Yesenia Cevallos, Paola Gabriela Vinueza-Naranjo, Deysi Inca, Cristian Vacacela Gomez

**Affiliations:** 1 Department of Chemistry, Universidad Técnica Particular de Loja, Loja 110160, Ecuador; 2 Universidad San Francisco de Quito IMNE, Diego de Robles s/n, Cumbayá, Quito 170901, Ecuador; 3 College of Engineering, Universidad Nacional de Chimborazo, Riobamba 060108, Ecuador; 4 ETEL Research Group, Faculty of Engineering and Applied Sciences, Networking and Telecommunications Engineering, Universidad de Las Américas (UDLA), Quito 170503, Ecuador; 5 INFN-Laboratori Nazionali di Frascati, Via E. Fermi 54, 00044 Frascati, Italy

**Keywords:** surface plasmon resonance, Kretschmann configuration, transfer matrix method, silicon nitride, black phosphorous, biosensors, malaria

## Abstract

This study presents a black phosphorus-based surface plasmon resonance (SPR) biosensor for malaria detection, integrating silicon nitride (Si_3_N_4_) and single-stranded DNA (ssDNA) to enhance sensitivity and molecular recognition. The biosensor configurations were optimized through numerical simulations, evaluating metal thickness, dielectric layer thickness, and the number of black phosphorus layers to achieve maximum performance. The optimized system (Opt-Sys_4_) exhibited high sensitivity (464.4°/RIU for early-stage malaria) and improved detection accuracy, outperforming conventional SPR sensors. Performance was assessed across malaria progression stages, demonstrating a clear resonance shift, increased attenuation, and enhanced biomolecular interactions. Key metrics, including the figure of merit, limit of detection, and comprehensive sensitivity factor, confirmed the sensor’s superior performance. Comparative analysis against state-of-the-art SPR biosensors further validated their capability for highly sensitive and specific malaria detection. These findings establish a promising plasmonic biosensing platform for early malaria diagnosis, potentially improving disease management in resource-limited settings.

## 1. Introduction

Malaria remains one of the most pressing global health challenges, disproportionately affecting tropical and subtropical regions [1]. Caused by Plasmodium parasites and transmitted through Anopheles mosquito bites [2], malaria leads to severe complications and death if not promptly diagnosed and treated. According to the World Health Organization (WHO), malaria caused an estimated 249 million cases and 608,000 deaths in 2022, with the highest burden in sub-Saharan Africa [3]. While control strategies such as insecticide-treated nets [4], vaccines [5], and chemoprevention [6] have reduced mortality rates, accurate and early detection remains a critical factor in malaria elimination efforts. Conventional diagnostic methods, including microscopy [7], rapid diagnostic tests (RDTs) [8], and polymerase chain reaction (PCR) [9] techniques, face significant limitations such as false negatives, reagent instability, high costs, and dependence on specialized personnel and infrastructure. These challenges necessitate the development of more sensitive, rapid, and cost-effective diagnostic technologies.

In recent years, optical biosensors have gained increasing attention for disease detection due to their high sensitivity, real-time monitoring capability, and label-free detection [10]. Among these, surface plasmon resonance (SPR) sensors have emerged as a powerful platform for biomolecular detection [11], offering superior performance over traditional diagnostic techniques. SPR is based on the excitation of surface plasmons—collective oscillations of conduction electrons—at a metal–dielectric interface when light is incident at a specific angle [12,13,14]. This resonance condition is highly sensitive to changes in the refractive index of the surrounding medium, allowing for the detection of biomolecular interactions in real time. The Kretschmann configuration is the most widely used SPR setup [15], where a thin metallic film (typically gold or silver) is deposited on a prism, enabling total internal reflection to excite surface plasmons. The binding of biomolecules, such as malaria biomarkers, induces shifts in the SPR resonance angle, which can be detected with high precision [16].

Despite their advantages, traditional SPR sensors still face limitations in terms of sensitivity, specificity, and stability. To overcome these challenges, researchers have explored the integration of two-dimensional (2D) nanomaterials [17] into SPR platforms. 2D materials, such as graphene [18], graphene nanoribbons arrays [19], molybdenum disulfide (MoS_2_) [20], and black phosphorus (BP) [21], offer unique electronic and optical properties that can enhance sensor performance. Among these, black phosphorus (BP) has recently emerged as a promising material for biosensing applications due to its layer-dependent bandgap, high surface-to-volume ratio, and strong light–matter interaction [22]. These properties make BP an excellent candidate for improving SPR sensitivity by amplifying the signal response and facilitating biomolecule immobilization.

Additionally, the incorporation of silicon nitride (Si_3_N_4_) as a dielectric layer can further enhance SPR sensor performance [23]. Silicon nitride is widely used in optical devices due to its high refractive index (n ≈ 2.0), excellent chemical stability, and low optical loss. When combined with black phosphorus, silicon nitride can optimize light confinement, reduce energy losses, and improve sensor resolution, making it a valuable component in next-generation biosensors [24]. With this in mind, this present study explores the potential of a black phosphorus-enhanced SPR biosensor, integrated with a silicon nitride dielectric layer, for malaria detection. By leveraging the unique optical properties of BP and the stability of Si_3_N_4_, we aim to develop a highly sensitive, rapid, and reliable diagnostic platform capable of detecting malaria biomarkers with greater accuracy than conventional methods. This approach holds promise for point-of-care applications, particularly in resource-limited settings, where early and efficient malaria diagnosis is crucial for disease control and treatment.

## 2. Materials and Methods

### 2.1. Theoretical Framework and Performance Metrics

The reflective intensity of the proposed *Nth*-layer sensor model is calculated using the TMM [20,25,26]. To summarize, the total reflection of the *Nth*-layer model can be expressed as (Equation (1)):

(1) $$R={\left|\frac{\left(M_{11}+M_{12}\;q_{N}\right)q_{1}-\left(M_{21}+M_{22}\;q_{N}\right)}{\left(M_{11}+M_{12}\;q_{N}\right)q_{1}+\left(M_{21}+M_{22}\;q_{N}\right)}\right|}^{2}$$

By using Equation (1), the reflectance as a function of the angle of incidence (SPR curve) can be calculated. Then, we now move on to the main performance metrics of the proposed sensors. The first parameter is the sensitivity enhancement regarding the baseline sensors after/before pathogen adsorption, denoted as (Equation (2)):

(2) $$∆S_{RI}^{after}=\frac{\left(S_{RI}^{after}-S_{RI}^{before}\right)}{S_{RI}^{before}}$$

However, the sensitivity to the refractive index change can be expressed as (Equation (3)):

(3) $$S_{RI}=\frac{∆\theta }{∆n}$$

Here, ∆θ represents the angle shift variation, and ∆n represents the refractive index variation.

The detection accuracy (*DA*) can be expressed in terms of ∆θ and the full width at half maximum (*FWHM*) of the SPR curve, as (Equation (4)):

(4) $$DA=\frac{∆\theta }{FWHM}$$

The Quality Factor (*QF*) can be expressed in terms of $S_{RI}$ and *FWHM*, as follows (Equation (5)):

(5) $$QF=\frac{S_{RI}}{FWHM}$$

The figure of merit (*FoM*) can be expressed as (Equation (6)):

(6) $$FoM=\frac{S_{RI}(1-R_{min})}{FWHM}$$

Here, $R_{min}$ represents the lowest normalized reflection value of the SPR curve.

The limit of detection (*LoD*) can be calculated as (Equation (7)):

(7) $$LoD=\frac{∆n}{∆\theta }\times 0.005{}^{\circ}$$

Finally, the comprehensive sensitivity factor (*CSF*) ratio can be calculated (Equation (8)) [27]:

(8) $$CSF=\frac{S_{RI}\times (R_{max}-R_{min})}{FWHM}$$

$R_{max}$ represents the maximum reflectance before resonance, typically at non-resonant wavelengths or angles. All computations in this investigation are performed with a data sampling of $3\times 10^{4}$ points.

### 2.2. Biosensor Configuration and Initial Parameters

Table S1 presents five SPR biosensor configurations (Sys_0_ to Sys_4_) with different material compositions and sensing media. Each system builds upon the Prism/Silver (P/Ag) base, progressively integrating materials to enhance SPR sensitivity. Sys_0_ (P/Ag/M_Blood_) uses plasma blood, serving as a biological reference. Sys_1_ (P/Ag/Stage_I_) represents the baseline system with normal erythrocytes before malaria infection. Sys_2_ (P/Ag/SN/Stage_I_) incorporates silicon nitride (Si_3_N_4_) to enhance optical properties. Sys_3_ (P/Ag/SN/BP/Stage_I_) adds black phosphorus (BP), which is known for improving SPR signal response. Sys_4_ (P/Ag/SN/BP/ssDNA/Stage_I_) includes single-stranded DNA (ssDNA), which is expected to make the system suitable for biomolecular recognition.

Table S2 lists the initial parameters adopted for the SPR biosensor before optimization, detailing the refractive index (RI) and thickness of each material at 633 nm. The prism (BK-7, P) has a refractive index of 1.5151 [20], serving as the optical coupling medium. The silver (Ag) layer, essential for SPR excitation, has a complex refractive index of 0.056253 + 4.2760i and a thickness of 55.0 nm [20]. Silicon nitride (Si_3_N_4_), used as a dielectric layer, has an RI of 2.0394 and a thickness of 5.00 nm [24]. Black phosphorus (BP), known for enhancing SPR sensitivity, has a refractive index of 3.5 + 0.01i with a thickness of 0.53 nm [27]. The sensing media include plasma blood (RI = 1.340) [16] and erythrocytes at Stage I (RI = 1.402) [16,28,29], both crucial for evaluating malaria detection. Therefore, these parameters define the initial structure of the SPR biosensor, providing the foundation for further optimization.

## 3. Results and Discussion

### 3.1. Selection of the Best Configurations

**Figure 1:**
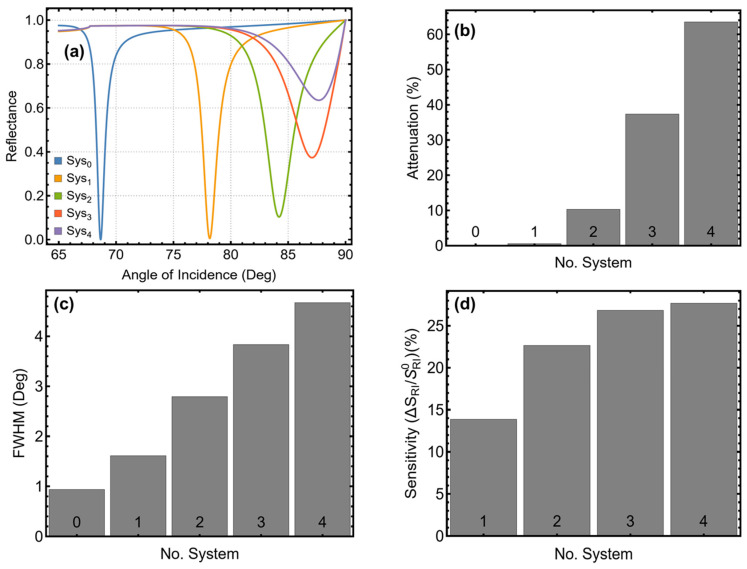
Performance analysis of the SPR biosensor configurations. (**a**) SPR reflectance curves for different configurations from Sys_0_ to Sys_4_. (**b**) Percentage of attenuation for different configurations. (**c**) Full width at half maximum (*FWHM*) for different configurations. (**d**) Sensitivity enhancement (%) for configurations Sys_1_–Sys_4_ compared to the reference configuration Sys_0_.

Figure 1 and Table S3 present the performance evaluation of the SPR biosensors for different configurations (Sys_0_ to Sys_4_) based on reflectance curves, attenuation percentage, full width at half maximum (*FWHM*), and sensitivity enhancement. The analysis focuses on the system behavior before and after transitioning from plasma blood (Sys_0_) to normal erythrocytes (Stage I), aiming to identify the most effective setup. The reflectance curves in Figure 1a indicate a significant shift in the SPR peak position as additional materials are incorporated. Table S3 quantifies this shift, showing that Sys_0_ (P/Ag/M_Blood_) exhibits an SPR peak at 68.65°, while Sys_1_ (P/Ag/Stage_I_) increases to 78.16°. With Si_3_N_4_ inclusion (Sys_2_), the peak shifts further to 84.20°, and Sys_3_ and Sys_4_, which incorporate BP, reach 87.08° and 87.74°, respectively. These results demonstrate that black phosphorus significantly alters the plasmonic response, enhancing the sensor’s ability to detect refractive index variations.

Figure 1b and Table S3 highlight the percentage of attenuation for each configuration. Sys_0_ has negligible attenuation (0.02%), acting as a reference. The inclusion of Si_3_N_4_ in Sys_2_ increases attenuation to 10.34%, while Sys_3_ (BP-based) reaches 37.34%, and Sys_4_ (BP with ssDNA) achieves the highest attenuation at 63.50%. The progressive increase in attenuation suggests that black phosphorus effectively enhances light–matter interaction. Figure 1c illustrates the *FWHM* change for each system, with values reported in Table S3. Sys_0_ exhibits the narrowest *FWHM* (0.94°), while Sys_1_ to Sys_4_ progressively widen, reaching 4.68° for Sys_4_. The increase in *FWHM* indicates greater plasmon broadening, which is characteristic of improved light absorption and sensor performance. Sensitivity enhancement, shown in Figure 1d, follows a similar trend. Sys_1_ provides a 13.85% improvement over Sys_0_, while Sys_2_ (Si_3_N_4_-based) reaches 22.65%. Notably, Sys_3_ and Sys_4_, both incorporating BP, achieve 26.84% and 27.70% enhancement, respectively, confirming that black phosphorus significantly boosts SPR sensitivity.

The progressive increase in resonance angle, attenuation, *FWHM*, and sensitivity observed can be attributed to the incremental addition of plasmonic and dielectric layers, which modify the optical properties of the SPR sensor. In particular, the introduction of Si_3_N_4_ enhances light confinement at the metal–dielectric interface by increasing the effective refractive index of the intermediate layer [23]. This results in a stronger evanescent field near the sensing region, leading to a shift in the resonance angle and improved plasmonic coupling efficiency. As well, Si_3_N_4_ acts as an optical buffer layer, reducing energy dissipation into the metal while maintaining strong plasmonic excitation. The increased refractive index at the interface leads to a higher effective wavevector, which requires a larger resonance angle for phase matching between the incident light and surface plasmon waves.

The further incorporation of 2D materials, particularly black phosphorus (BP), significantly alters the plasmonic response due to its high refractive index and anisotropic optical properties, which increase attenuation and *FWHM* by enhancing light–matter interactions. Additionally, the presence of ssDNA in Sys_4_ further improves sensitivity by facilitating biorecognition and molecular interaction, making the system more responsive to refractive index variations. These cumulative effects explain the observed trend in performance metrics, where each additional layer contributes to enhanced SPR signal modulation and biosensing efficiency.

Based on these findings, Sys_4_ (P/Ag/SN/BP/ssDNA/Stage_I_) (Figure S1a) emerges as the most effective system. The incorporation of ssDNA further optimizes molecular interaction, making it the preferred choice for biosensing applications. However, Sys_3_ (P/Ag/SN/BP/Stage_I_) (Figure S1b) is retained for comparison, as it provides strong SPR performance without the additional functionalization layer.

### 3.2. Metal Thickness Optimization

**Figure 2:**
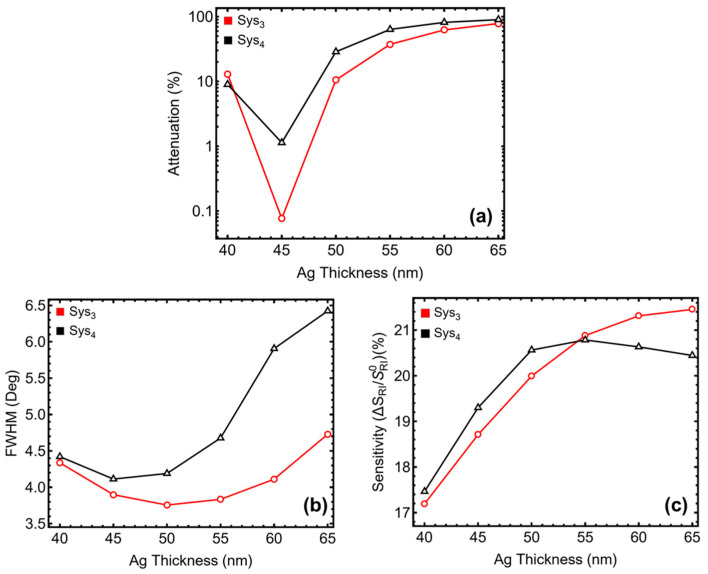
Performance analysis of Sys_3_ and Sys_4_ configurations with varying silver (Ag) thicknesses from 40 to 65 nm. (**a**) Percentage of attenuation for each configuration, the *y*-axis-log scale is considered. (**b**) Full width at half maximum (*FWHM*) for each configuration. (**c**) Sensitivity enhancement (%) for Sys_3_ and Sys_4_, relative to the baseline systems constructed with initial parameters.

The results in Figure 2 and Table S4 demonstrate the impact of silver thickness variation on the SPR peak position, attenuation, FHWM, and sensitivity enhancement for Sys_3_ and Sys_4_. The attenuation values indicate that thinner silver layers (40–45 nm) result in lower energy loss, whereas thicker layers (50–65 nm) exhibit higher attenuation, with Sys_4_ reaching 89.81% at 65 nm (Figure 2a). While high attenuation can improve plasmonic coupling, excessive losses may reduce signal clarity and stability. The SPR peak position shifts progressively with increasing thickness, stabilizing around 87.49° for Sys_3_ and 87.42° for Sys_4_ at 65 nm. This shift reflects the sensor’s increased refractive index sensitivity due to enhanced plasmonic interactions.

For *FWHM* (Figure 2b), a thinner silver layer (40–45 nm) results in narrower resonance curves due to lower ohmic losses and better-defined plasmonic oscillations. At 45 nm, *FWHM* remains relatively small (3.90° for Sys_3_ and 4.11° for Sys_4_), meaning the resonance peak is well-defined, leading to improved detection accuracy. However, as the thickness increases beyond 50 nm, plasmon damping and radiative losses intensify, causing broader resonance curves. This effect is particularly pronounced at 65 nm, where *FWHM* expands to 4.73° for Sys_3_ and 6.42° for Sys_4_, indicating reduced spectral resolution and poorer signal discrimination for small refractive index changes.

The sensitivity enhancement follows an increasing trend up to 55 nm, where it reaches 20.88% for Sys_3_ and 20.78% for Sys_4_ before stabilizing. Initially, increasing silver thickness improves plasmonic confinement, enhancing the sensor’s response to refractive index variations. However, beyond 55 nm, the rate of sensitivity improvement diminishes as excessive thickness leads to weaker field penetration into the sensing medium. This saturation effect arises because a thicker metallic layer confines more energy within the metal rather than allowing interaction with the analyte, thereby limiting additional gains in sensitivity.

To further remark, the non-monotonic behavior of attenuation and *FWHM* with increasing silver thickness in Figure 2 can be explained by the trade-off between plasmonic confinement and ohmic losses. At thinner silver layers (40–45 nm), lower attenuation is observed due to reduced energy dissipation, leading to well-defined plasmonic oscillations. As the thickness increases beyond this range, the plasmonic coupling is enhanced, but excessive metal thickness results in higher radiative and ohmic losses, which broaden the resonance curve and increase attenuation. Similarly, the sensitivity enhancement in Sys_4_ first rises with silver thickness as stronger plasmonic fields improve refractive index detection, but beyond 55 nm, the response stabilizes due to the diminishing interaction between the evanescent field and the sensing medium. These trends align with the selection of 45 nm as the optimal thickness, ensuring a balance between sensitivity, spectral resolution, and plasmonic efficiency.

The observed trends in *FWHM* and sensitivity justify the selection of 45 nm as the optimal silver thickness. At this point, the sensor maintains a sharp resonance peak, ensuring high spectral resolution while still benefiting from significant sensitivity enhancement without excessive damping effects [30].

### 3.3. Silicon Nitride Thickness Optimization

**Figure 3:**
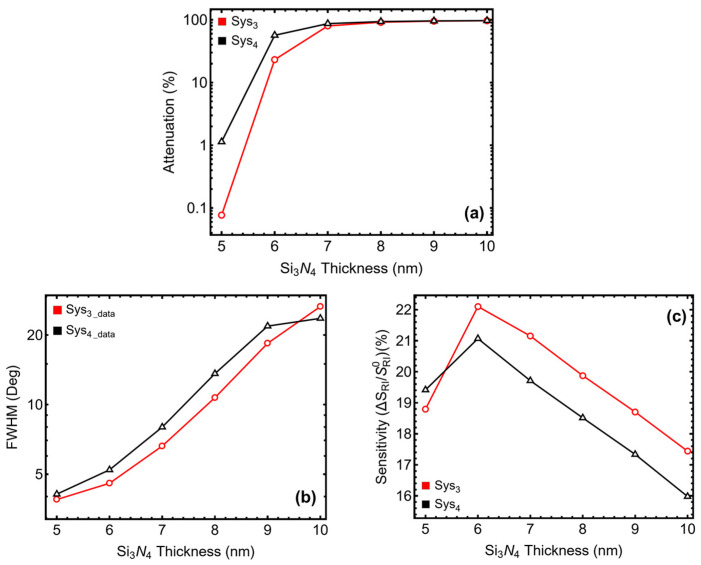
Performance analysis of Sys_3_ and Sys_4_ configurations with varying silicon nitride thicknesses from 5 to 10 nm. (**a**) Percentage of attenuation for each configuration, the *y*-axis-log scale is considered. (**b**) Full width at half maximum (*FWHM*) for each configuration. (**c**) Sensitivity enhancement (%) for Sys_3_ and Sys_4_, relative to the baseline systems constructed with initial parameters and optimized silver thickness value.

The results in Figure 3 and Table S5 show the impact of silicon nitride (Si_3_N_4_) thickness variation from 5 to 10 nm. The attenuation percentage, shown in Figure 3a and Table S5, increases significantly as Si_3_N_4_ thickness increases. For Sys_3_, attenuation rises from 0.08% at 5 nm to 96.72% at 10 nm, while for Sys_4_, it increases from 1.14% to 97.07% over the same range. The increase beyond 6 nm suggests that thicker Si_3_N_4_ layers enhance light absorption but can also introduce excessive damping, reducing plasmonic efficiency. The SPR peak position follows a progressive shift with increasing Si_3_N_4_ thickness. In Sys_3_, the peak moves from 85.51° at 5 nm to 84.54° at 10 nm, showing a slight redshift beyond 7 nm. A similar trend is observed in Sys_4_, where the peak moves from 86.59° at 5 nm to 84.10° at 10 nm, indicating that excessive thickness can alter the resonance condition, potentially affecting detection accuracy.

The *FWHM* values, shown in Figure 3b, enlarges notably as Si_3_N_4_ thickness increases. For Sys_3_, it grows from 3.89° at 5 nm to 26.54° at 10 nm, while for Sys_4_, it rises from 4.10° to 23.60°. This trend confirms that thicker layers cause broader resonance curves, reducing spectral resolution and making it harder to distinguish small refractive index changes. The sensitivity enhancement, presented in Figure 3c, follows a nonlinear trend. It increases up to 6 nm, reaching 22.10% for Sys_3_ and 21.07% for Sys_4_, but then declines beyond this thickness, falling to 17.44% and 15.98% at 10 nm, respectively. The initial increase suggests that Si_3_N_4_ enhances field confinement, improving the sensor’s response. However, after 7 nm, excessive thickness limits field penetration into the sensing medium, reducing its effect.

From these results, we selected 5 nm as the optimal Si_3_N_4_ thickness, as it ensures sharp resonance peaks, minimal broadening, and stable sensitivity enhancement while avoiding excessive damping effects. This balance maintains high spectral resolution and optimal detection performance in biosensing applications.

### 3.4. Two-Dimensional Nanolayers Optimization

The results in Figure S2 and Table S6 show the effect of black phosphorus (BP) layers from one (L1) to six (L6). The attenuation percentage, shown in Figure S2a and Table S6, remains low at L1 but increases significantly beyond L2. For Sys_3_, attenuation rises from 0.08% at L1 to 97.23% at L6, while for Sys_4_, it increases from 1.14% to 97.42%. This increase indicates that while BP enhances plasmonic interaction, excessive layers lead to strong damping and energy loss. The SPR peak position shifts downward with increasing BP layers. In Sys_3_, it moves from 85.51° at L1 to 83.42° at L6, and in Sys_4_, from 86.59° at L1 to 82.90° at L6.

The *FWHM* values, presented in Figure S2b, increase as the number of BP layers grows. For Sys_3_, it starts at 3.89° at L1 and expands to 34.97° at L6, while in Sys_4_, it rises from 4.10° to 59.89°. This broadening confirms that additional BP layers contribute to higher energy dissipation and spectral widening, reducing sensor resolution and precision. The sensitivity enhancement, as shown in Figure S2c, follows a peak-trough trend. It increases up to L2, reaching 22.12% for Sys_3_ and 20.76% for Sys_4_, but declines beyond this, dropping to 15.89% and 14.33% at L6, respectively. This behavior suggests that while a few BP layers improve the plasmonic field interaction, excessive thickness reduces field penetration into the sensing medium, limiting further sensitivity gains.

From these results, we selected a single BP layer (L1) as the optimal choice since it maintains low attenuation, sharp resonance peaks, and high spectral resolution without excessive broadening or damping effects.

From the fabrication viewpoint, there are several methods to synthesize single-layer black phosphorus (BP) for SPR sensor applications, including liquid-phase exfoliation [31], mechanical exfoliation [32], and pulsed laser deposition [33]. However, chemical vapor deposition (CVD) is currently the most promising approach due to its ability to produce high-quality, large-area monolayers with controlled thickness directly on sensor substrates [34]. Thus, to fabricate the SPR sensor from BK7 glass to the BP monolayer, the following steps are suggested:

- The BK7 prism serves as the optical coupling element.
- A 55 nm silver (Ag) layer could be deposited via thermal evaporation or sputtering.
- A 5 nm Si_3_N_4_ layer could be deposited using plasma-enhanced chemical vapor deposition (PECVD) [35].
- Red phosphorus or phosphine gas (PH_3_) could be heated under an inert atmosphere (Ar/N_2_).
- The BP monolayer can be directly grown on Si_3_N_4_/Au using a low-pressure CVD system at 400–600 °C.
- Controlled growth time and carrier gas flow regulate monolayer thickness.
- Immediately after growth, the BP monolayer can be encapsulated with hexagonal boron nitride (hBN) or atomic-layer-deposited (ALD) Al_2_O_3_ to prevent degradation [36]. While encapsulation enhances BP stability, it may slightly modify the plasmonic response of the sensor, which should be considered in experimental evaluations.
- The SPR chip could be cleaned under inert conditions to avoid oxidation.

### 3.5. ssDNA Thickness Optimization

**Figure 4:**
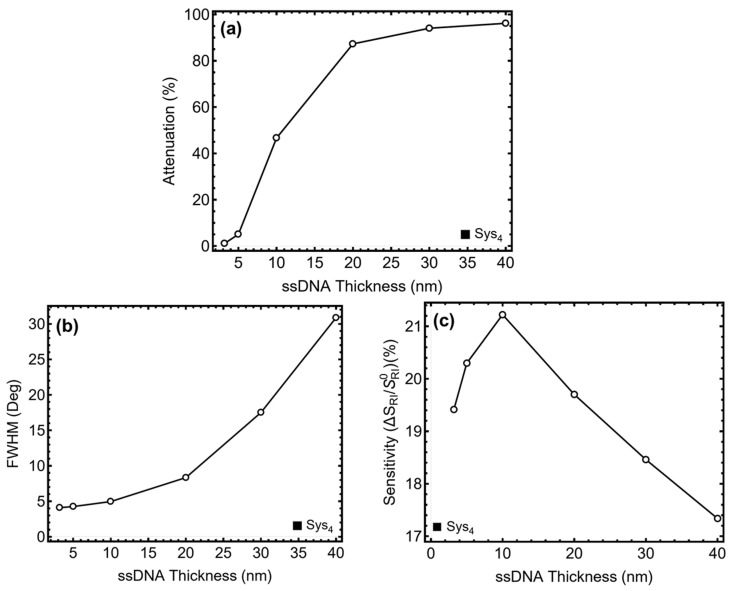
Performance analysis of Sys_4_ configuration with varying the ssDNA layer thickness from 3.2 nm to 50 nm. (**a**) Percentage of attenuation for each configuration. (**b**) Full width at half maximum (*FWHM*) for each configuration. (**c**) Sensitivity enhancement (%), relative to the baseline system constructed with initial parameters and optimized silver/silicon nitride/black phosphorous thickness values.

The results in Figure 4 and Table S7 illustrate the impact of ssDNA thickness variation for Sys_4_. Since Sys_4_ is the only configuration incorporating ssDNA, the analysis focuses on how varying the thickness from 3.2 nm to 40 nm influences sensor performance, particularly in terms of plasmonic coupling and biomolecular interaction. The attenuation percentage, shown in Figure 4a and Table S7, increases with ssDNA thickness, indicating stronger interaction with the plasmonic field. At 3.2 nm, attenuation is low (1.14%), suggesting weak coupling. As the thickness increases to 5 nm, attenuation reaches 5.03%, indicating improved plasmonic response. However, beyond 10 nm (46.67%), attenuation rises steeply, exceeding 87% at 20 nm and reaching 96.19% at 40 nm. This confirms that while a moderate ssDNA layer enhances plasmonic sensitivity, excessive thickness introduces high optical losses, leading to plasmon damping and reduced sensor efficiency.

The SPR peak position follows a controlled redshift with increasing ssDNA thickness, as seen in Table S7. At 3.2 nm, the peak is at 86.59°, shifting to 87.23° at 5 nm due to biomolecular layer formation. This shift remains within a reasonable range for effective sensing. However, beyond 10 nm, the peak position begins to decline, reaching 85.08° at 40 nm, indicating that excessive thickness alters the effective refractive index of the sensing region, potentially affecting the accuracy of biomolecule detection. The *FWHM* values, presented in Figure 4b, remain relatively small for thin ssDNA layers, ensuring high spectral resolution. At 3.2 nm, *FWHM* is 4.10°, slightly increasing to 4.25° at 5 nm. However, as thickness increases, *FWHM* expands significantly, reaching 30.87° at 40 nm, indicating a broader resonance curve and reduced signal clarity. The steep increase beyond 10 nm suggests that excessive ssDNA thickness compromises spectral precision, making it difficult to distinguish minor refractive index changes. The sensitivity enhancement, shown in Figure 4c, follows a peak-trough pattern. At 5 nm, sensitivity reaches 20.30%, approaching the maximum value of 21.22% observed at 10 nm. However, beyond 10 nm, sensitivity starts declining, dropping to 17.34% at 40 nm. This confirms that while moderate ssDNA thickness improves molecular recognition and plasmonic coupling, excessive thickness limits the field penetration, reducing further sensor enhancement. Considering these observations, 5 nm was selected as the optimal ssDNA thickness. Compared to 3.2 nm, it enhances plasmonic coupling while avoiding the high attenuation and excessive broadening observed at 10 nm and beyond.

Table S8 presents the optimized structural parameters for Sys_3_ and Sys_4_, detailing the refractive index (RI) and thickness values of each material layer. For Opt-Sys_3_, the sensor consists of a BK7 prism (RI = 1.5151), a 45 nm silver (Ag) layer, a 5 nm silicon nitride (Si_3_N_4_) dielectric layer, and a single-layer black phosphorus (BP) film (0.53 nm). Opt-Sys_4_ follows the same structure but includes a 5 nm ssDNA functionalization layer (RI = 1.462) for biomolecular interaction. The refractive index values of malaria-infected erythrocytes decrease as the infection progresses [16,28,29]: 1.395 for the ring stage (II), 1.381 for the trophozoite stage (III), and 1.371 for the schizont stage (IV).

### 3.6. Malaria Detection

**Figure 5:**
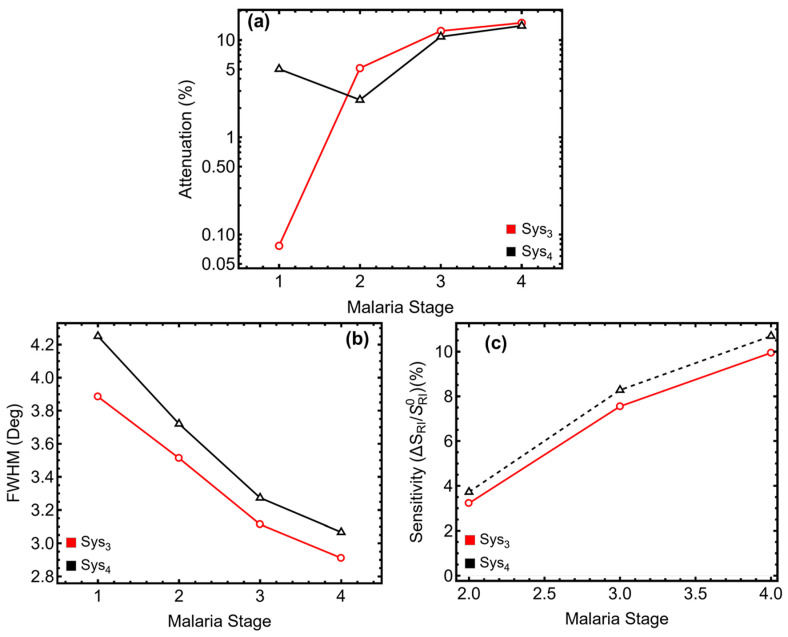
Performance analysis of optimized Sys_3_ and Sys_4_ configurations with varying the Malaria stage from Normal (I) to Schizont (IV). (**a**) Percentage of attenuation for each configuration, *y*-axis-log scale is considered. (**b**) Full width at half maximum (*FWHM*) for each configuration. (**c**) Sensitivity enhancement (%) for Sys_3_ and Sys_4_, relative to the optimized systems, taking as a sensing medium the Normal (I) stage.

The results in Figure 5 and Table S9 present the performance of the optimized Sys_3_ and Sys_4_ configurations in detecting different malaria stages (Ring II, Trophozoite III, and Schizont IV). These findings directly address the study’s primary objective: evaluating the sensor’s ability to track malaria progression through refractive index variations. Then, the SPR peak position, shown in Table S9, shifts significantly as the malaria stage progresses. For Opt-Sys_3_, the peak moves from 85.51° in Normal (I) to 77.01° in Schizont (IV), while in Opt-Sys_4_, it shifts from 87.23° to 77.90°. This redshift confirms that as erythrocytes undergo structural changes due to infection, the sensor effectively detects the corresponding refractive index decrease, making it suitable for malaria diagnostics. The attenuation percentage, depicted in Figure 5a, increases with malaria progression. In Opt-Sys_3_, attenuation rises from 0.08% in Normal (I) to 15.02% in Schizont (IV), while in Opt-Sys_4_, it increases from 5.03% to 13.99%. This suggests that the structural and biochemical modifications in infected erythrocytes enhance plasmonic absorption.

The *FWHM* trend, observed in Figure 5b, decreases with malaria progression. For Opt-Sys_3_, it narrows from 3.89° at Normal (I) to 2.91° at Schizont (IV), while for Opt-Sys_4_, it reduces from 4.25° to 3.07°. The narrowing resonance curve indicates that as malaria progresses, the sensor maintains high resolution and sharper detection capabilities, essential for accurate stage differentiation. The sensitivity enhancement, presented in Figure 5c, follows an increasing trend. In Opt-Sys_3_, it rises from 3.23% in Ring (II) to 9.95% in Schizont (IV), while in Opt-Sys_4_, it increases from 3.73% to 10.70%. The higher sensitivity in Opt-Sys_4_ confirms that the ssDNA layer enhances biomolecular recognition, improving malaria detection efficiency. The optimized systems, particularly Opt-Sys_4_, provide high sensitivity and reliable differentiation of malaria stages, making them suitable for clinical diagnostic applications.

In practical applications, the resonance angle of the proposed SPR sensor naturally shifts to lower values as malaria progresses, making measurements more accessible. While the initial resonance angle for Sys_4_ with Normal (I) reaches approximately 87°, it gradually decreases to 77° for Sys_4_ and 76° for Sys_3_ in the Schizont (IV) stage, aligning with the typical operational range for SPR setups using a BK7 prism. Although high resonance angles (∼87°) approach grazing incidence, they remain experimentally measurable with a well-configured SPR setup. To achieve accurate detection at these angles, goniometric rotation stages with fine angular resolution allow precise control of the incident light. Additionally, collimated laser beams, optimized beam alignment, and high-sensitivity photodetectors ensure reliable reflectivity measurements. In practical implementations, adjustments to the detection system can help maintain signal stability even at steep angles. Since the primary objective of this sensor is to monitor malaria progression, the natural shift toward lower resonance angles in later infection stages (∼76–77°) further supports the practical feasibility of the measurement setup.

### 3.7. Performance Sensing Metrics

Table 1 presents the key SPR biosensor performance metrics for different malaria stages using the optimized sensor configurations Opt-Sys_3_ and Opt-Sys_4_. The evaluated parameters include resonance angle shift variation (Δ*θ*), sensitivity (S), detection accuracy (*DA*), and quality factor (*QF*), which are essential for determining the sensor’s effectiveness in distinguishing malaria progression. The resonance angle shift (Δ*θ*) increases as malaria progresses. In Opt-Sys_3_, it rises from 2.76° in Ring (II) to 8.51° in Schizont (IV), while in Opt-Sys_4_, it follows the same trend, increasing from 3.25° to 9.33°. The higher Δ*θ* in Opt-Sys_4_ suggests that the ssDNA layer improves the biomolecular interaction, leading to stronger refractive index variations.

The sensitivity (S), measured in °/RIU, shows a decreasing trend as malaria advances. In Opt-Sys_3_, it starts at 394.00°/RIU for Ring (II) and drops to 274.43°/RIU in Schizont (IV). A similar trend is observed in Opt-Sys_4_, where sensitivity declines from 464.38°/RIU to 301.08°/RIU. This behavior indicates that as malaria progresses, the refractive index contrast between the stages becomes less pronounced, requiring highly sensitive detection systems. The detection accuracy (*DA*) improves with disease progression. For Opt-Sys_3_, *DA* increases from 0.785 at Ring (II) to 2.922 at Schizont (IV), while for Opt-Sys_4_, it rises from 0.873 to 3.043. The higher *DA* in Opt-Sys_4_ further confirms that the functionalization layer enhances specificity, leading to better differentiation between malaria stages.

The quality factor (*QF*), representing the sharpness of the SPR curve, is higher in early infection stages and decreases with progression. In Opt-Sys_3_, *QF* drops from 112.15 RIU^−1^ at Ring (II) to 94.27 RIU^−1^ at Schizont (IV), while in Opt-Sys_4_, it decreases from 124.82 RIU^−1^ to 98.17 RIU^−1^. This suggests that while later-stage infections lead to broader resonance curves, the optimized system retains strong resolution and detection efficiency. As noted, these results confirm that Opt-Sys_4_ outperforms Opt-Sys_3_ in resonance shift, sensitivity, and detection accuracy, making it the most effective sensor configuration for malaria detection and stage differentiation.

Table 2 presents additional SPR biosensor performance metrics for different malaria stages, specifically evaluating the figure of merit (*FoM*), limit of detection (*LoD*), and comprehensive sensitivity factor (*CSF*) for Opt-Sys_3_ and Opt-Sys_4_. These parameters further quantify the sensor’s effectiveness in malaria stage differentiation. In Opt-Sys_3_, *FoM* drops from 106.38 RIU^−1^ in Ring (II) to 80.11 RIU^−1^ in Schizont (IV). Similarly, in Opt-Sys_4_, it starts at 121.79 RIU^−1^ for Ring (II) and decreases to 84.43 RIU^−1^ in Schizont (IV). The higher *FoM* in Opt-Sys_4_ across all stages confirms that ssDNA functionalization enhances signal precision and plasmonic response, making it more effective for malaria detection.

The limit of detection (*LoD*), which quantifies the smallest refractive index variation the sensor can detect, increases as malaria progresses. In Opt-Sys_3_, *LoD* rises from 1.27 × 10^−5^ for Ring (II) to 1.82 × 10^−5^ for Schizont (IV), while in Opt-Sys_4_, it follows the same trend, increasing from 1.08 × 10^−5^ to 1.66 × 10^−5^. Lower LoD values in Opt-Sys_4_ indicate higher sensitivity, confirming that this configuration provides better detection capabilities for early-stage malaria infections.

The comprehensive sensitivity factor (*CSF*) decreases with malaria progression. In Opt-Sys_3_, it declines from 103.82 in Ring (II) to 77.84 in Schizont (IV), while in Opt-Sys_4_, it starts higher at 118.94 and reduces to 82.14. The higher *CSF* values in Opt-Sys_4_ suggest that it maintains greater sensitivity and accuracy across all malaria stages. All these findings confirm that Opt-Sys_4_ consistently outperforms Opt-Sys_3_ in resolution, detection limit, and overall sensitivity, making it the most effective sensor configuration for tracking malaria progression.

### 3.8. State-of-the-Art Comparison

Table 3 provides a comparative analysis of the proposed SPR biosensors (Sys_2_, Sys_3_, and Sys_4_) with state-of-the-art configurations reported in the literature. The metric of interest is sensitivity (S, in °/RIU). The previously reported SPR biosensors, incorporating graphene, transition metal dichalcogenides (TMDs), and MXenes, exhibit sensitivity values ranging from 194.0°/RIU to 258.3°/RIU. Notably, the highest sensitivity among these studies was achieved using an Au/MXene/Au/Graphene configuration with 258.3°/RIU for the Trophozoite (III) stage [16], which represents one of the best plasmonic structures in the literature.

However, our proposed sensors outperform these state-of-the-art designs, particularly Sys_3_ and Sys_4_, which integrate black phosphorus (BP) and ssDNA functionalization. For instance, Sys_3_ (Ag/Si_3_N_4_/BP) reaches 394.0°/RIU for the Ring (II) stage, 307.5°/RIU for Trophozoite (III), and 274.4°/RIU for Schizont (IV), significantly surpassing previous works. The introduction of ssDNA in Sys_4_ further enhances the sensitivity, achieving 464.4°/RIU for the Ring (II) stage, 343.9°/RIU for Trophozoite (III), and 301.1°/RIU for Schizont (IV). These values confirm that black phosphorus and molecular functionalization play a key role in improving plasmonic interaction and biomolecular recognition. For comparison purposes, we show the results for Sys_1_ and Sys_2_ (Figure S3). Additionally, we emphasize that all results presented in the current work were obtained by fixing the refractive index of BP, which remains stable up to five layers, as supported by in [27,41]

## 4. Conclusions

In summary, this work presents a black phosphorus-enhanced SPR biosensor optimized for malaria detection by integrating Si_3_N_4_ and ssDNA layers. Systematic parameter optimization demonstrated that a 45 nm silver layer, 5 nm silicon nitride, and a single-layer black phosphorus film provided the best plasmonic response. The functionalization with 5 nm ssDNA further increased molecular recognition, enabling more sensitive detection of malaria biomarkers. The optimized biosensor (Opt-Sys_4_) exhibited higher resonance shifts, increased sensitivity, and improved detection accuracy across malaria progression stages. Compared to state-of-the-art designs, the proposed sensor achieved a higher figure of merit, lower detection limit, and enhanced sensitivity, confirming its superior diagnostic potential. These results indicate that the black phosphorus-based SPR biosensor is a powerful candidate for malaria diagnosis, offering a highly sensitive, cost-effective, and real-time detection platform suitable for clinical and point-of-care applications. Future work should focus on experimental validation and integration into portable diagnostic devices to facilitate realistic implementation.

## Figures and Tables

**Table 1:** Numerical values of the SPR biosensor performance metrics for different malaria stages. Δ*θ* represents the resonance angle shift variation, *S* is the sensitivity, *DA* is the detection accuracy, and *QF* is the quality factor.

Malaria Stage	∆θ	*S* (°/RIU)	*DA*	*QF* (*RIU*^−1^)
Opt-Sys_3_				
Ring (II)	2.758	394.000	0.785	112.148
Trophozoite (III)	6.457	307.460	2.073	98.751
Schizont (IV)	8.507	274.430	2.922	94.265
Opt-Sys_4_				
Ring (II)	3.251	464.381	0.873	124.818
Trophozoite (III)	7.222	343.937	2.206	105.061
Schizont (IV)	9.333	301.075	3.043	98.172

**Table 2:** Numerical values of the additional SPR biosensor performance metrics for different malaria stages. *FoM* represents the figure of merit, *LoD* is the limit of detection, and *CSF* is the comprehensive sensitivity factor.

Malaria Stage	*FoM* (*RIU*^−1^)	*LoD* (10^−5^)	*CSF*
Opt-Sys_3_			
Ring (II)	106.375	1.269	103.822
Trophozoite (III)	86.519	1.626	84.279
Schizont (IV)	80.111	1.822	77.843
Opt-Sys_4_			
Ring (II)	121.791	1.076	118.939
Trophozoite (III)	93.689	1.454	91.316
Schizont (IV)	84.434	1.660	82.135

**Table 3:** Comparison with state-of-the-art SPR biosensors.

Configuration	*S* (°/*RIU*)	Ref.
TiO_2_/Ag/MoSe/Graphene	194.0	[37]
Au/PtSe_2_/Graphene	200.0	[38]
TiO_2_/ZnO/Au/MoS_2_/GO	210.8	[39]
Rh/Ag/Si/Graphene	220.0	[40]
Au/MXene/Au/Graphene (Trophozoite (III))	258.3	[16]
Ag (Sys_1_)	190.95 (II)	This work
	175.95 (III)	
	167.63 (IV)	
Ag/Si_3_N_4_ (Sys_2_)	302.62 (II)	This work
	254.48 (III)	
	233.20 (IV)	
Ag/Si_3_N_4_/Black Phosphorus (Sys_3_)	394.0 (II)	This work
	307.5 (III)	
	274.4 (IV)	
Ag/Si_3_N_4_/Black Phosphorus/ssDNA (Sys_4_)	464.4 (II)	This work
	343.9 (III)	
	301.1 (IV)	

## Data Availability

The original contributions presented in the study are included in the article/Supplementary Materials. Further inquiries can be directed at the corresponding author.

## Supplementary Materials

The following supporting information can be downloaded at: https://www.mdpi.com/article/10.3390/s25072068/s1, Figure S1: Schematic representation of the proposed SPR biosensor configurations. (a) Sys_3_ and (b) Sys_4_. Figure S2: Performance analysis of Sys_3_ and Sys_4_ configurations with varying the number of black phosphorous layers from 1 (L1) to 6 (L6). b (a) Percentage of attenuation for each configuration, *y*-axis-log scale is considered. (b) Full width at half maximum (*FWHM*) for each configuration. (c) Sensitivity enhancement (%) for Sys_3_ and Sys_4_, relative to the baseline systems constructed with initial parameters and optimized silver/silicon nitride thickness values. Figure S3: SPR curves for (a) Sys_2_ and (b) Sys_3_ at different Malaria stages. (c) Sensitivity to refractive index change for the different Malaria stages. Table S1: Configurations of the SPR biosensors evaluated in this study, using different materials and sensing media. The “Full Name” column describes the structure from bottom to top. Table S2: Initial parameters adopted in the SPR biosensor configuration before optimization. The refractive index (RI) and thickness values for each material used in the sensor’s construction are shown at 633 nm. Table S3: Summary of numerical performance metrics of SPR Peak Position, Attenuation (%), Full Width at Half Maximum (*FWHM*), and Sensitivity Enhancement (%) for Systems Sys_0_ through Sys_4_. Table S4: Summary of numerical performance metrics of SPR Peak Position, Attenuation (%), Full Width at Half Maximum (*FWHM*), and Sensitivity Enhancement (%) by changing the silver thickness for Sys_3_ and Sys_4_. Table S5: Summary of numerical performance metrics of SPR Peak Position, Attenuation (%), Full Width at Half Maximum (*FWHM*), and Sensitivity Enhancement (%) by changing the silicon nitride thickness for Sys_3_ and Sys_4_. Table S6: Summary of numerical performance metrics of SPR Peak Position, Attenuation (%), Full Width at Half Maximum (*FWHM*), and Sensitivity Enhancement (%) by increasing the number of graphene layers in Sys_3_ and Sys_4_. Table S7: Summary of numerical performance metrics of SPR Peak Position, Attenuation (%), Full Width at Half Maximum (*FWHM*), and Sensitivity Enhancement (%) by changing the ssDNA layer thickness for Sys_4_. Table S8: Summary of optimized parameters for Sys_3_ and Sys_4_. Additionally, the refractive index values for different Malaria stage have been reported. Table S9: Summary of numerical performance metrics of SPR Peak Position, Attenuation (%), Full Width at Half Maximum (*FWHM*), and Sensitivity Enhancement (%) at different Malaria stages for optimized Sys_3_ and Sys_4_.
