# Supplementary material for: Black Phosphorous-Based Surface Plasmon Resonance Biosensor for Malaria Diagnosis
Source: Sensors (Basel). 2025 Mar 26;25(7):2068. doi: 10.3390/s25072068 (PMC11991473; doi:10.3390/s25072068)
Supplement: Supplementary file 1 [file sensors-25-02068-s001.zip › sensors-3517428-supplementary.pdf]

Black Phosphorous-Based SPR Biosensor for Malaria Diagnosis

Talia Tene[1,*], Yesenia Cevallos[2,3], Paola Gabriela Vinueza-Naranjo[4,3], Deysi Inca[3], Cristian Vacacela Gomez[5,*]

[1] Department of Chemistry, Universidad Técnica Particular de Loja, Loja 110160, Ecuador

[2] Universidad San Francisco de Quito IMNE, Diego de Robles s/n, Cumbayá, Quito 170901, Ecuador

[3] College of Engineering, Universidad Nacional de Chimborazo, Riobamba 060108, Ecuador

[4] Faculty of Engineering and Applied Sciences, Networking and Telecommunications Engineering, ETEL Research Group, Universidad de Las Américas (UDLA), Quito 170503, Ecuador.

[5] INFN-Laboratori Nazionali di Frascati, Via E. Fermi 54, 00044 Frascati, Italy

Correspondence: tbtene@utpl.edu.ec (T.T.) & cristianisaac.vacacelagomez@utpl.edu.ec (C.V.G.)

Supplementary Figures

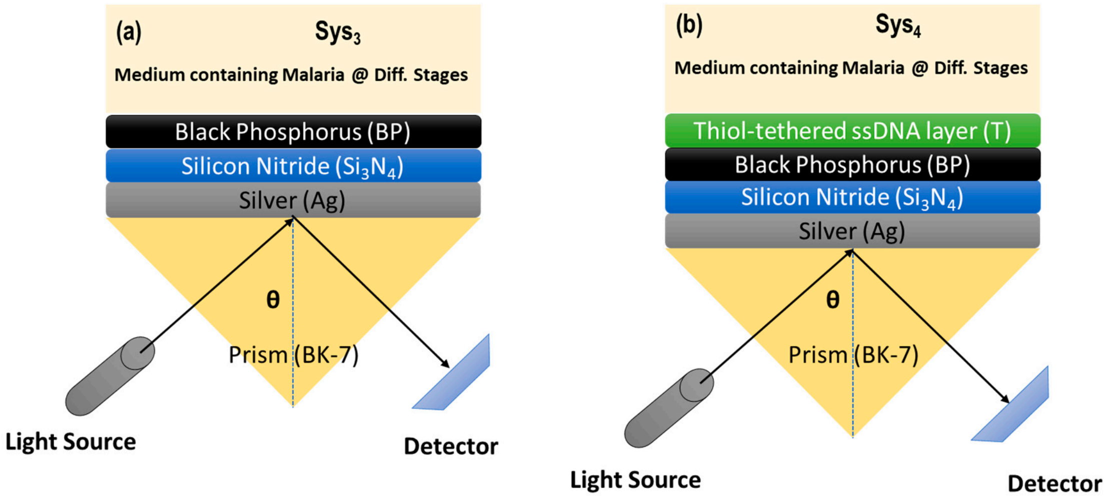

Figure S1. Schematic representation of the proposed SPR biosensor configurations. (a) Sys_3 and (b) Sys_4.

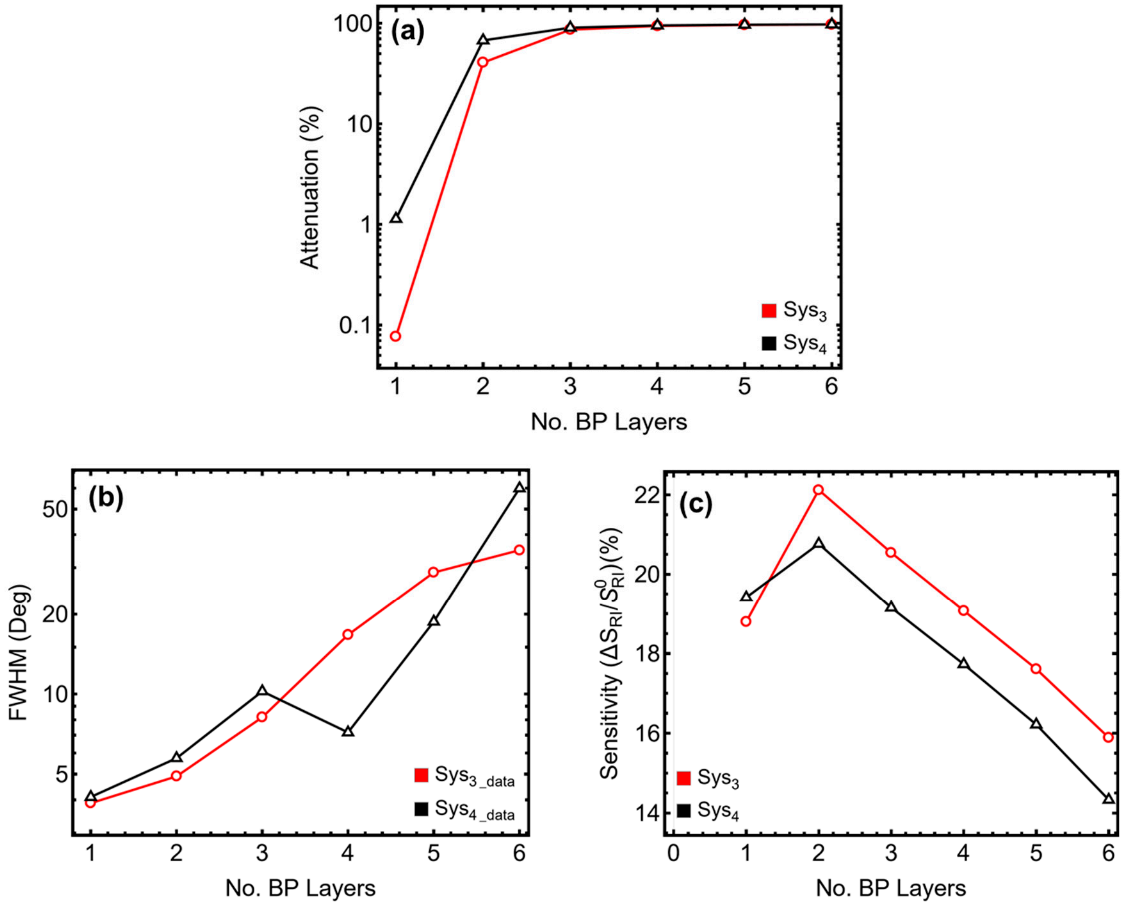

Figure S2. Performance analysis of Sys_3 and Sys_4 configurations with varying the number of black phosphorous layers from 1 (L1) to 6 (L6). b (a) Percentage of attenuation for each configuration, y-axis-log scale is considered. (b) Full width at half maximum (FWHM) for each configuration. (c) Sensitivity enhancement (%) for Sys_3 and Sys_4, relative to the baseline systems constructed with initial parameters and optimized silver/silicon nitride thickness values.

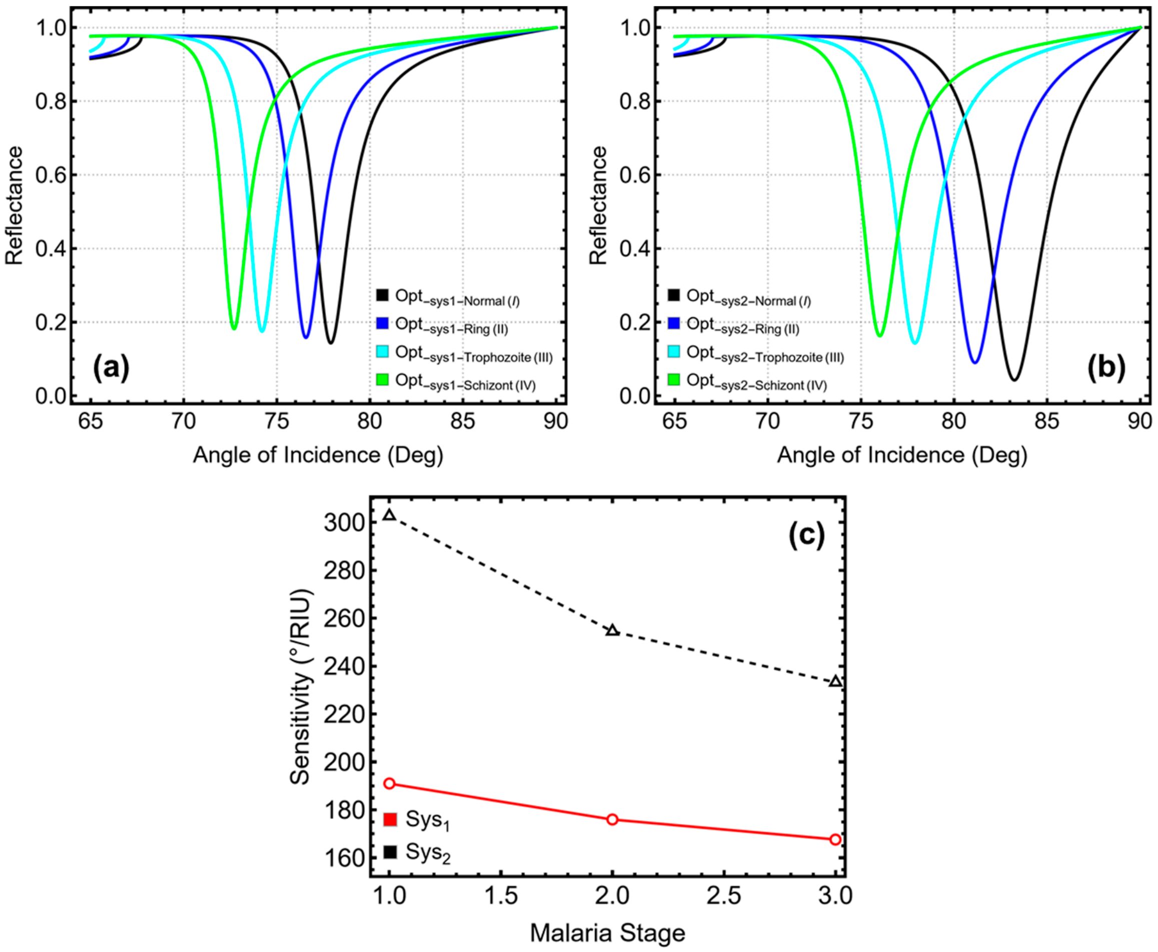

Figure S3. SPR curves for (a) Sys_2 and (b) Sys_3 at different Malaria stages. (c) Sensitivity to refractive index change for the different Malaria stages.

Supplementary Tables

Table S1. Configurations of the SPR biosensors evaluated in this study, using different materials and sensing media. The "Full Name" column describes the structure from bottom to top.

Sys No.	Code	Full Name	Nick Name
0	Sys_0	Prism/Silver/Plasma Blood	$P/Ag/M_{Blood}$
1	Sys_1	Prism/Silver/Normal (I)	P/Ag/Stage I
2	Sys_2	Prism/Silver/Si_3N_4/Normal (I)	P/Ag/SN/Stage I
3	Sys_3	Prism/Silver/Si_3N_4/Black Phosphorus/Normal (I)	P/Ag/SN/BP/Stage I
4	Sys_4	Prism/Silver/Si_3N_4/ Black Phosphorus /ssDNA/Normal (I)	P/Ag/SN/BP/ssDNA/Stage I

Table S2. Initial parameters adopted in the SPR biosensor configuration before optimization. The refractive index (RI) and thickness values for each material used in the sensor's construction are shown at 633 nm.

Material	Refractive Index	Thickness (nm)	Ref.
BK-7 (P)	1.5151	---	[20]
Silver (Ag)	0.056253 + 4.2760 *i*	55.0	[20]
Si_3N_4 (SiN)	2.0394	5.00	[24]
Black Phosphorus (BP)	3.5 + 0.01 *i*	0.53	[27]
Plasma blood	1.340	---	[16]
Normal (I) stage (erythrocytes)	1.402	---	[16, 28, 29]

Table S3. Summary of numerical performance metrics of SPR Peak Position, Attenuation (%), Full Width at Half Maximum (FWHM), and Sensitivity Enhancement (%) for Systems Sys_0 through Sys_4.

Sys No.	Code	SPR Peak position	Attenuation (%)	FWHM	Enhancement (%)
0	Sys_0	68.651	0.019	0.937	0.0
1	Sys_1	78.162	0.551	1.611	13.854
2	Sys_2	84.201	10.336	2.792	22.650
3	Sys_3	87.078	37.343	3.833	26.840
4	Sys_4	87.744	63.495	4.676	27.698

Table S4. Summary of numerical performance metrics of SPR Peak Position, Attenuation (%), Full Width at Half Maximum (FWHM), and Sensitivity Enhancement (%) by changing the silver thickness for Sys_3 and Sys_4.

**Thickness (nm)**	**SPR Peak position**	**Attenuation (%)**	**FWHM**	**Enhancement (%)**
		**Sys_3**		
40	84.421	12.982	4.336	17.193
45	85.514	0.077	3.898	18.709
50	86.438	10.549	3.755	19.993
55	87.078	37.344	3.833	20.880
60	87.388	62.377	4.111	21.312
65	87.490	78.245	4.729	21.452
		**Sys_4**		
40	85.258	8.988	4.421	17.465
45	86.590	1.135	4.113	19.301
50	87.506	28.695	4.189	20.563
55	87.674	63.495	4.676	20.784
60	87.556	81.546	5.906	20.632
65	87.420	89.811	6.424	20.444

Table S5. Summary of numerical performance metrics of SPR Peak Position, Attenuation (%), Full Width at Half Maximum (FWHM), and Sensitivity Enhancement (%) by changing the silicon nitride thickness for Sys_3 and Sys_4.

**Thickness (nm)**	**SPR Peak position**	**Attenuation (%)**	**FWHM**	**Enhancement (%)**
		**Sys_3**		
5	85.514	0.077	3.885	18.796
6	87.894	23.135	4.568	22.102
7	87.210	79.796	6.619	21.152
8	86.291	91.692	10.715	19.875
9	85.447	95.253	18.417	18.703
10	84.537	96.716	26.541	17.439
		**Sys_4**		
5	86.590	1.135	4.099	19.415
6	87.789	56.665	5.221	21.068
7	86.805	86.882	7.992	19.711
8	85.936	93.638	13.609	18.513
9	85.082	96.018	21.854	17.335
10	84.098	97.072	23.600	15.978

Table S6. Summary of numerical performance metrics of SPR Peak Position, Attenuation (%), Full Width at Half Maximum (FWHM), and Sensitivity Enhancement (%) by increasing the number of graphene layers in Sys_3 and Sys_4.

**No. Layers**	**SPR Peak position**	**Attenuation (%)**	**FWHM**	**Enhancement (%)**
		**Sys_3**		
L1	85.514	0.077	3.887	18.796
L2	87.909	40.667	4.902	22.123
L3	86.772	86.392	8.179	20.542
L4	85.714	93.985	16.699	19.074
L5	84.662	96.292	28.794	17.612
L6	83.422	97.226	34.974	15.889
		**Sys_4**		
L1	86.590	1.135	4.099	19.414
L2	87.570	67.544	5.737	20.766
L3	86.399	90.346	10.238	19.152
L4	85.369	95.087	7.172	17.731
L5	84.272	96.726	18.722	16.218
L6	82.904	97.422	59.887	14.331

Table S7. Summary of numerical performance metrics of SPR Peak Position, Attenuation (%), Full Width at Half Maximum (FWHM), and Sensitivity Enhancement (%) by changing the ssDNA layer thickness for Sys_4.

**Thickness (nm)**	**SPR Peak position**	**Attenuation (%)**	**FWHM**	**Enhancement (%)**
		**Sys_4**		
3.2	86.590	1.135	4.100	19.414
5	87.230	5.032	4.254	20.297
10	87.901	46.667	4.959	21.222
20	86.798	87.293	8.335	19.701
30	85.898	94.042	17.507	18.460
40	85.084	96.187	30.871	17.338

Table S8. Summary of optimized parameters for Sys_3 and Sys_4. Additionally, the refractive index values for different Malaria stage have been reported.

Material	Refractive Index (RI)	Thickness (nm)
	**Opt-Sys_3**	
BK7 (P)	1.5151	---
Ag	0.056253 + 4.2760 i	45.0
Si_3N_4 (SiN)	2.0394	5.0
Black Phosphorus (BP)	3.5 + 0.01 i	0.53*L (L=1)
	**Opt-Sys_4**	
BK7 (P)	1.5151	---
Ag	0.056253 + 4.2760 i	45.0
Si_3N_4 (SiN)	2.0394	5.0
Black Phosphorus (BP)	3.5 + 0.01 i	0.53*L (L=1)
ssDNA	1.462	5.0
	**Malaria Stage**	
Ring (II)	1.395	---
Trophozoite (III)	1.381	---
Schizont (IV)	1.371	---

Table S9. Summary of numerical performance metrics of SPR Peak Position, Attenuation (%), Full Width at Half Maximum (FWHM), and Sensitivity Enhancement (%) at different Malaria stages for optimized Sys_3 and Sys_4.

**Thickness (nm)**	**SPR Peak position**	**Attenuation (%)**	**FWHM**	**Enhancement (%)**
**Opt-Sys_3**				
Normal (I)	85.514	0.076	3.885	0.0
Ring (II)	82.756	5.147	3.513	3.225
Trophozoite (III)	79.057	12.386	3.113	7.550
Schizont (IV)	77.006	15.015	2.911	9.948
**Opt-Sys_4**				
Normal (I)	87.230	5.032	4.251	0.0
Ring (II)	83.979	2.425	3.720	3.727
Trophozoite (III)	80.007	10.823	3.274	8.280
Schizont (IV)	77.897	13.995	3.067	10.699
